# The m^6^A-methylated mRNA pattern and the activation of the Wnt signaling pathway under the hyper-m^6^A-modifying condition in the keloid

**DOI:** 10.3389/fcell.2022.947337

**Published:** 2022-10-03

**Authors:** Can-Xiang Lin, Zhi-Jing Chen, Qi-Lin Peng, Ke-Rong Xiang, Du-Qing Xiao, Ruo-Xi Chen, Taixing Cui, Yue-Sheng Huang, Hong-Wei Liu

**Affiliations:** ^1^ Department of Plastic Surgery of the First Affiliated Hospital of Jinan University, Institute of New Technology of Plastic Surgery of Jinan University, Key Laboratory of Regenerative Medicine of Ministry of Education, Guangzhou, China; ^2^ The Research Center of Medicine, Sun Yat-Sen Memorial Hospital, Sun Yat-Sen University, Guangzhou, China; ^3^ Department of Thoracic Surgery, The First Affiliated Hospital of Jinan University, Guangzhou, China; ^4^ Department of Cell Biology and Anatomy, University of South Carolina School of Medicine, Columbia, SC, United States; ^5^ Department of Wound Repair, Institute of Wound Repair and Regeneration Medicine, Southern University of Science and Technology Hospital, Southern University of Science and Technology School of Medicine, Shenzhen, China

**Keywords:** keloid, m^6^A modification, fibroblasts, the Wnt signaling pathway, RNA sequencing

## Abstract

**Purpose:** The present study was carried out to investigate the global m^6^A-modified RNA pattern and possible mechanisms underlying the pathogenesis of keloid.

**Method:** In total, 14 normal skin and 14 keloid tissue samples were first collected on clinics. Then, three samples from each group were randomly selected to be verified with the Western blotting to determine the level of methyltransferase and demethylase. The total RNA of all samples in each group was isolated and subjected to the analysis of MeRIP sequencing and RNA sequencing. Using software of MeTDiff and *htseq-*count, the m^6^A peaks and differentially expressed genes (DEGs) were determined within the fold change >2 and *p-*value < 0.05. The top 10 pathways of m^6^A-modified genes in each group and the differentially expressed genes were enriched by the Kyoto Encyclopedia of Genes and Genomes signaling pathways. Finally, the closely associated pathway was determined using the Western blotting and immunofluorescence staining.

**Results:** There was a higher protein level of WTAP and Mettl3 in the keloid than in the normal tissue. In the keloid samples, 21,020 unique m^6^A peaks with 6,573 unique m^6^A-associated genetic transcripts appeared. In the normal tissue, 4,028 unique m^6^A peaks with 779 m^6^A-associated modified genes appeared. In the RNA sequencing, there were 847 genes significantly changed between these groups, transcriptionally. The genes with m^6^A-methylated modification and the upregulated differentially expressed genes between two tissues were both mainly related to the Wnt signaling pathway. Moreover, the hyper-m^6^A-modified Wnt/*β*-catenin pathway in keloid was verified with Western blotting. From the immunofluorescence staining results, we found that the accumulated fibroblasts were under a hyper-m^6^A condition in the keloid, and the Wnt/*β*-Catenin signaling pathway was mainly activated in the fibroblasts.

**Conclusion:** The fibroblasts in the keloid were under a cellular hyper-m^6^A-methylated condition, and the hyper-m^6^A-modified highly expressed Wnt/*β*-catenin pathway in the dermal fibroblasts might promote the pathogenesis of keloid.

## Introduction

As one of the benign fibro-proliferative tumors, keloid influences about 11 million patients physically or psychologically around the world (Boahene et al., 2018). Patients with keloid usually suffer from pain, itching, bleeding, ulceration, and even movement disorder (Wang et al., 2021). Several methods including traditional surgery, radiotherapy, and hormone therapy have been applied to the treatment of keloid currently (Lee et al., 2019; Lee et al., 2020). However, there are still some limitations to these therapies as the site where the keloid was surgically removed is prone to recurrence (Lee et al., 2019). It is difficult to achieve a complete cure for this disease. Moreover, recent studies have indicated that patients with keloids have a higher risk of developing various cancers than normal individuals (Lu et al., 2021). In a matched, population-based regression analysis study (17,401 patients with keloid vs. 69,604 controls), Ying-Yi et al. have shown that the overall cancer risk was 1.49-fold higher in the keloid group than controls (Lu et al., 2021). Henry Ford Health System suggested a link between keloids and increased risk of being diagnosed with breast cancer, particularly among African Americans (Zhang et al., 2019). Regarding the potential role in the correlation between keloid and cancer, it is of high importance to have a good understanding of the genomic signature of keloid.

N^6^-methyladenosine (m^6^A) methylation, a methylating modification occurring in the N^6^-position of adenosine, is one of the most common internal mRNA epitranscriptomic modifications in eukaryotic cells (Liu and Gregory, 2019). Recently, accumulating evidence revealed that m^6^A methylation can directly affect the progress of translation, degradation, splicing, outputting, and folding of mRNA (He et al., 2019). The modification is catalyzed by m^6^A methyltransferases of methyltransferase-like 3 and 14 (METTL3 and METTL14) and regulated by the co-factor, Wilm’s tumor 1-associated protein (WTAP) (Liu et al., 2014). Conversely, the progress of demethylation is mediated by the demethylases including the fat mass and obesity-associated protein (FTO) (Jia et al., 2011) and AlkB homolog 5 (ALKBH5) (Zheng et al., 2013). This mechanism maintains the dynamic balance of human m^6^A modification. Additionally, m^6^A-binding proteins, such as YTHDF1/2/3, mainly work as the “readers” to recognize the methylation of RNA (Li et al., 2021). For the past few years, more and more evidence has shown that m^6^A modification is related to the development of various diseases. For instance, m^6^A methylation can influence the transformation of a skin phenotype: Xi et al. (2020) presented the crosstalk between m^6^A modification and Wnt signaling pathway, indicating that the basal epidermal Wnt^hi^ progenitor was unable to progress to form hair follicles without m^6^A modification. Studies revealed that the fibrosis-related genes have hyper-m^6^A conditions and higher mRNA levels in hypertrophic scars than in normal skin (Liu et al., 2021). As for the molecular mechanism, the aberrant signal changes of the Wnt/*β*-catenin pathway can regulate the proliferation and migration of tumor cells and the activation of fibroblast in disease conditions (Dees and Distler, 2013; Zhang et al., 2019; Dahlmann et al., 2021). Moreover, S100A4 is the direct target gene of this pathway (Stein et al., 2006). As the levels of Wnt signaling are known to profoundly impact fate outcomes and proper tissue morphogenesis, the aberrant activation of the Wnt/*β*-catenin signaling pathway was also found in the disease of keloids (Lee et al., 2017). However, to the best of our knowledge, the mRNA m^6^A methylation profile of keloid has not been studied. Also, the modification of m^6^A methylation in the Wnt/*β*-catenin/S100A4 pathway remains unknown in keloid.

In this study, 14 keloid samples and 14 normal skin (NS) samples were collected on clinics. Western blotting (WB) test determined that the proteins of WTAP and METTL3 in the keloid group were higher than those in the normal group. Subsequently, the highly different m^6^A methylation patterns between the keloid and normal skin were first presented and analyzed with methylated RNA immunoprecipitation sequencing (MeRIP-seq) and RNA sequencing (RNA-seq). The methylated modification of the Wnt/*β*-catenin pathway in the keloid was indicated to be closely associated with the pathogenesis of keloid. The conjoint analysis showed that the Wnt/*β*-catenin signaling pathway in keloid was under the hyper-m^6^A methylation condition with its transcriptional level higher than in the normal skin. Consistently, the proteins of the Wnt/*β*-catenin pathway in the keloid group were higher than those in the normal skin group. In conclusion, the formation of keloid is closely associated with the cellular hyper-m^6^A-methylated condition, and the m^6^A-modified highly expressed Wnt/*β*-catenin pathway plays a role in the pathogenesis of keloid.

## Materials and methods

### Patients and samples

This study was approved by the Ethics Committee of The First Affiliated Hospital of Jinan University, and informed consent was obtained from all patients and unaffected individuals.

In total, 14 keloid samples were collected from 14 patients at the Department of Plastic Surgery at The First Affiliated Hospital of Jinan University (Table 1). In addition, 14 individuals without personal or family history of keloid were recruited from the Department of Thoracic Surgery and general surgery at The First Affiliated Hospital of Jinan University. Normal skin samples were obtained from these volunteers (Table 1). After being isolated, the samples were divided into two parts: one was immediately frozen and preserved in liquid nitrogen for the molecular analysis of MeRIP-seq, RNA-seq, and Western blotting test. The other part was stored in 4% formalin for the pathological change analysis of immunofluorescence staining.

**TABLE 1:** Information of each sample.

	Sex	Age	Biopsy site	Duration of the lesion	Etiology	Previous treatment
Keloid						
1	F	21	Upper limb	10 years	After injection	Topical corticosteroid
2	F	49	Chest	5 years	Acne	No treatment
3	M	9	Chest	3 years	Rupture of the cyst	No treatment
4	F	31	Face	1 year	Unknown	Unknown
5	F	31	Armpits	15 years	After operation	Topical corticosteroid
6	F	5	Knee	6 months	Empyrosis	Topical corticosteroid
7	M	14	Knee	3 months	Trauma	No treatment
8	F	10	Upper limb	1 year	After operation	Topical corticosteroid
9	M	8	Abdomen	2 years	After operation	Topical corticosteroid
10	F	14	Upper limb	5 months	Trauma	No treatment
11	M	15	Abdomen	2 years	After operation	Unknown
12	F	65	Earlobe	2 years	Unknown	Unknown
13	M	19	Abdomen	19 years	Congenital anomaly	Unknown
14	F	49	Abdomen	2 years	After operation	No treatment
Normal Skin						
1	M	60	Chest	—	—	—
2	M	41	Chest	—	—	—
3	F	63	Chest	—	—	—
4	F	45	Chest	—	—	—
5	F	51	Chest	—	—	—
6	F	63	Chest	—	—	—
7	M	27	Chest	—	—	—
8	F	55	Abdomen	—	—	—
9	F	54	Abdomen	—	—	—
10	F	65	Thigh	—	—	—
11	F	27	Abdomen	—	—	—
12	F	72	Chest	—	—	—
13	M	34	Chest	—	—	—
14	M	23	Abdomen	—	—	—

Abbreviations: M, male; F, female.

### MeRIP-seq and RNA-seq

The MeRIP-seq service was performed by OE Biotech Inc. (Shanghai, China). In short, total RNA was extracted from the keloid group and the NS group by using the TRIzol ® reagent (RNAiso Plus, Takara, 9109). Total RNA from 14 keloid samples (equal amount of RNA from each sample) was mixed to set as the keloid RNA pool, and the NS RNA pool was established using the similar method. Approximately 10 μg of double poly-A selected RNA was yielded from 400 μg total RNA in each biological replicate for m^6^A-seq starts. Fragmentation buffer (10 mM ZnCl2 and 10 mM Tris–HCl, pH7.0) was used to fragment poly-A RNA. By following the manufacturer’s instructions, m^6^A RNA immunoprecipitation was performed with anti-m^6^A (Synaptic Systems, Cat. No. 202 003). Both the input samples without immunoprecipitation (IP) for RNA-seq and the m^6^A IP samples for MeRIP-seq were together used to establish a library by utilizing the Illumina TrueSeq Stranded mRNA. To ensure the library quality, the fragment sizes of each individual library were verified by using an Agilent Bioanalyzer 2100.

### Sequencing data analysis

Raw data (raw reads) were first processed using Trimmomatic (Bolger et al., 2014) software. Clean data (clean reads) were obtained by removing reads containing adapter and ploy-N. Then, the clean reads were mapped to the reference genome using HISAT2 (Kim et al., 2015) with default parameters. The Guitar (Cui et al., 2016) R package and deepTools (Ramirez et al., 2014) software were used to evaluate the data quality of the clean data. The m^6^A-enriched peaks in each m^6^A immunoprecipitation sample were identified using MeTDiff (Cui et al., 2018) software with the corresponding input samples serving as a control. The differential peaks were detected using MeTDiff with parameters (FRAGMENT_LENGTH = 200, PEAK_CUTOFF_PVALUE = 0.01, DIFF_PEAK_CUTOFF_FDR = 0.05, and PEAK_CUTOFF_FDR = 0.05), after which, differential peaks were annotated by ChIPseeker. The relative strength of m^6^A peak sets was presented with the value of log2 fold change. The commonly shared, keloid-unique, or normal skin-unique m^6^A peaks sets were clustered with a heatmap through an R package of *pheatmap.* Gene Ontology (GO) enrichment and Kyoto Encyclopedia of Genes and Genomes (KEGG) pathway enrichment analyses were performed by the differentially methylated protein-coding genes. MEME (Bailey et al., 2009) was used to detect the sequence motif, after which, the compared reads can be visualized in the Integrative Genomics Viewer (IGV) to visually show the expression of targeted mRNA.

### Western blotting

Tissues were homogenized in RIPA lysis buffer containing a 50× protease inhibitor cocktail. Homogenates were then centrifuged at 19,392 g for 15 min to remove cell debris. The supernatant was collected, and total protein concentrations were measured using a protein assay kit (Enhance6d BCA Protein Assay Kit, Beyotime). A volume of 10 μg of the protein was electrophoretically separated in an SDS-PAGE gel (10% Tris–HCl) and transferred to 4.5-μm PVDF membranes. The membranes were blocked with 5% skim milk for 1 h and then incubated with rabbit anti-Mettl3 (1:1,000, ab195352, Abcam), rabbit anti-Mettl14 (1:1,000, ab220031, Abcam), rabbit anti-WTAP (1:1,000, ab195380, Abcam), rabbit anti-β-catenin (1:1,000, #8480, Cell Signaling Technology), rabbit anti-Wnt3 (1:1,000, #2721, Cell Signaling Technology), rabbit anti-S100A4 (1:1,000, ab124805, Abcam), rabbit anti-FTO (1:1,000, ab124892, Abcam), rabbit anti-ALKBH5 (1:1,000, ab195377, Abcam), and mouse anti-GAPDH (1:1,000, ab8245, Abcam) overnight at 4°C room temperature. The membranes were then washed in PBS-T (3 times for 10 min each) and incubated with the anti-rabbit secondary antibody (1:1,000, ab288151, Abcam) or anti-mouse secondary antibody (1:1,000, ab150113, Abcam) at room temperature for 1 h. Bands were visualized by ECL and quantitated using ImageJ software.

### Immunofluorescence staining

Tissues of normal skin and keloid were fixed in formalin for 48 h, embedded, and sectioned at a thickness of 4 μm. Then, the sections were deparaffinized, rehydrated, and blocked with phosphate-buffered saline (PBS) or 2% fetal bovine serum (FBS). Sections were incubated overnight at 4°C with specific primary antibody rabbit anti-S100A4 (1:500, Abcam, ab124805) and rabbit anti-WTAP (1:500, Abcam, ab195380) and then incubated with the secondary antibody goat anti-rabbit IgG (1:1,000, Abcam, ab15007) at 37°C for 30 min in dark. Subsequently, the sections were washed with PBS three times (5 min each time). Finally, the sections were counterstained with DAPI (1 μg/ml, Abcam, ab285390-5 mg) to allow visualization of the cell nucleus.

### Statistical analysis

Experiments were run at least three times, and representative data were presented as the mean ± SD. Statistical analysis was performed using GraphPad Prism 8.0 software. Student’s t-tests were performed between the keloid group and the normal skin group. A value of *p* < 0.05 was considered statistically significant.

## Results

### The overview of m^6^A modification in the keloid group and the NS group

**FIGURE 1:**
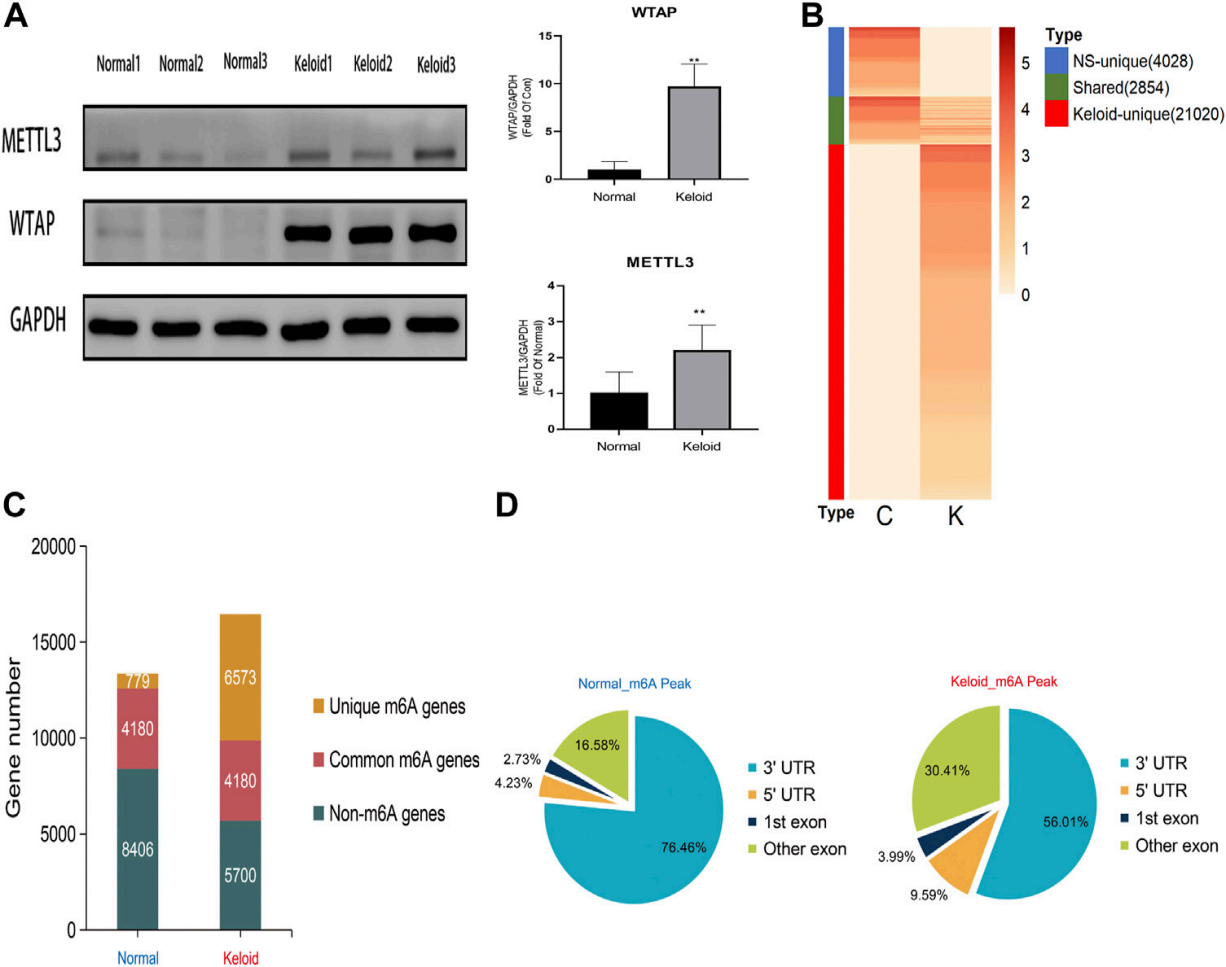
Transcriptome wide of MERIP-seq and the distribution of m^6^A peaks in the keloid tissue and in the normal skin tissue. **(A)** Protein level of the METTL3 and WTAP methyltransferases in two types of tissues. *n* = 3 samples were randomly selected from each group in the WB experiments, *p < 0.05. **(B)** Signal heatmaps of unique and shared m^6^A peaks between the keloid and normal skin. The signal was presented with the value of log2 fold change of the distinct m^6^A peak sets. **(C)** Summary of m^6^A-modified genes identified in both groups. **(D)** Distribution of m^6^A peaks in the genic regions of the two groups.

The Western blotting results showed that the proteins of METTL3 and WTAP in keloid were significantly higher than those in normal skin (Figure 1A, *p* < 0.05). But there is no significant change in METTL14, FTO, and ALKBH5 between these two groups (data are not shown), which indicates that the keloid is under a hypermethylated condition. In the RNA sequencing, we obtained 49853648 and 43208668 raw reads from keloid and NS samples, respectively. After removing the adapter, ploy-N, and low-quality reads, 47014732 (94.31%) and 39277827 (90.90%) reads of two groups were uniquely mapped to the reference genome by using HISAT2.

In keloid, a total of 23,874 m^6^A peaks were identified, while there were only 6,882 m^6^A peaks identified in the NS group (Figure 1B). Compared with the NS group, 21,020 new peaks appeared in the keloid group (Figure 1B). There were 2,854 commonly shared m^6^A peaks, corresponding to 4,180 gene transcripts (Figures 1B,C) between the two groups. The epitranscriptomic genome profile indicated the divergency of overall m^6^A modification patterns between the two groups. From the pie graphs, it is obvious to know that there are four positions of the m^6^A peak in the RNA transcript, namely, 5′-untranslated regions (5′-UTR), 3′-untranslated regions (3′-UTR), the first exon, and other exons. The distribution patterns of the keloid group and NS group showed similar trends. The peaks were enriched in the 3′-UTR (56.01% in the keloid group and 76.46% in the NS group) and the exon region apart from the first exon (30.41% and 16.58%, respectively), followed by the 5′-UTR (9.59% and 4.23%, respectively) (Figure 1D). Last, only 3.99% in the keloid group and 2.73% in the NS group m^6^A peaks were enriched in the first exon (Figure 1D).

### The landscape and pathway enrichments of m^6^A-modified transcripts in the keloid group and NS group

**FIGURE 2:**
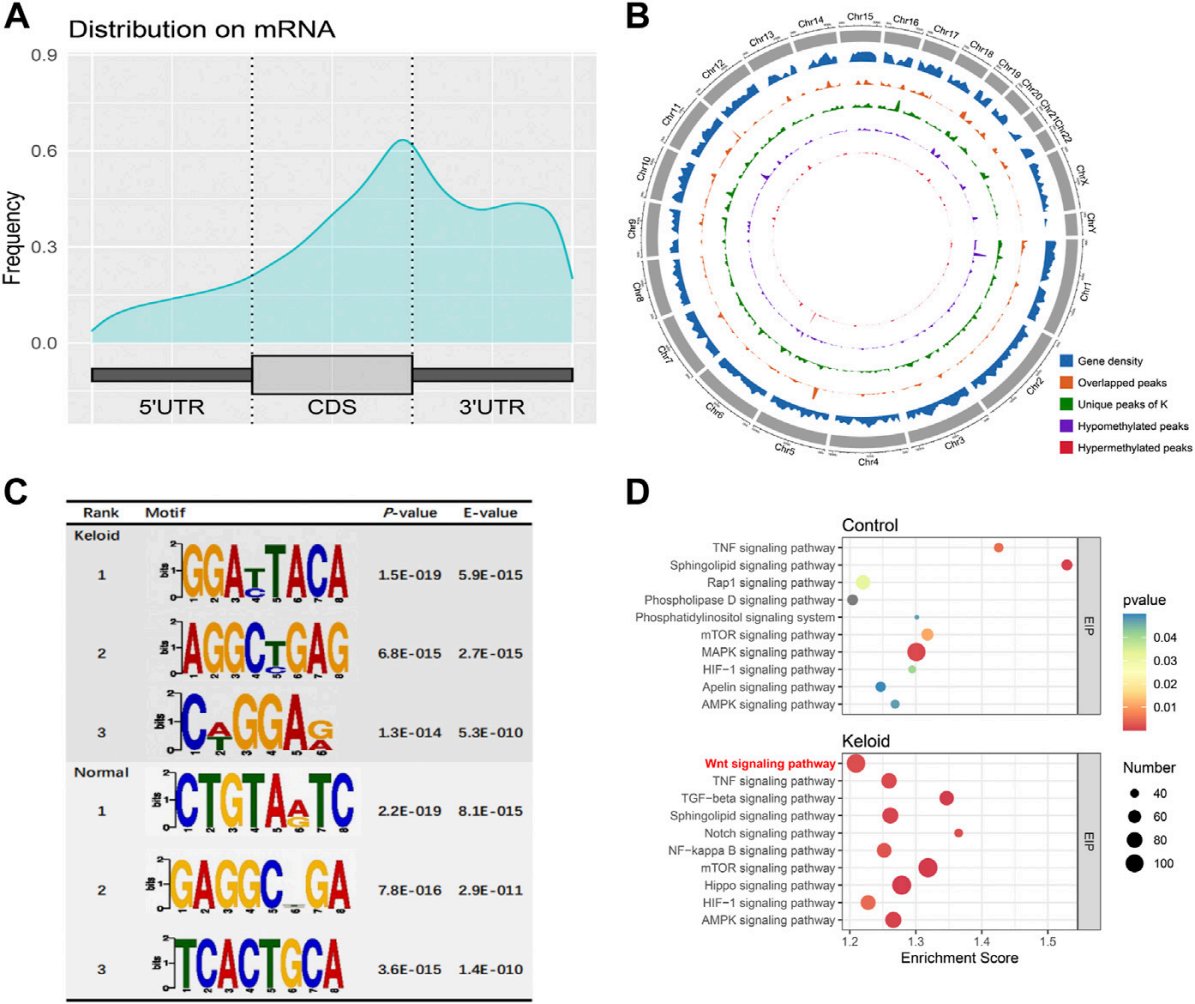
m^6^A-modified transcript profile and the associated pathway in two groups. **(A)** Distribution density of m^6^A peaks in the 5′-UTR, coding sequence (CDS), and 3′-UTR regions. **(B)** Density distribution of the m^6^A peaks on the 24 chromosomes. **(C)** Top three sequence motifs in two tissues. **(D)** Top 10 significantly enriched pathways of m^6^A-methylated genes in keloid and NS groups.

**FIGURE 3:**
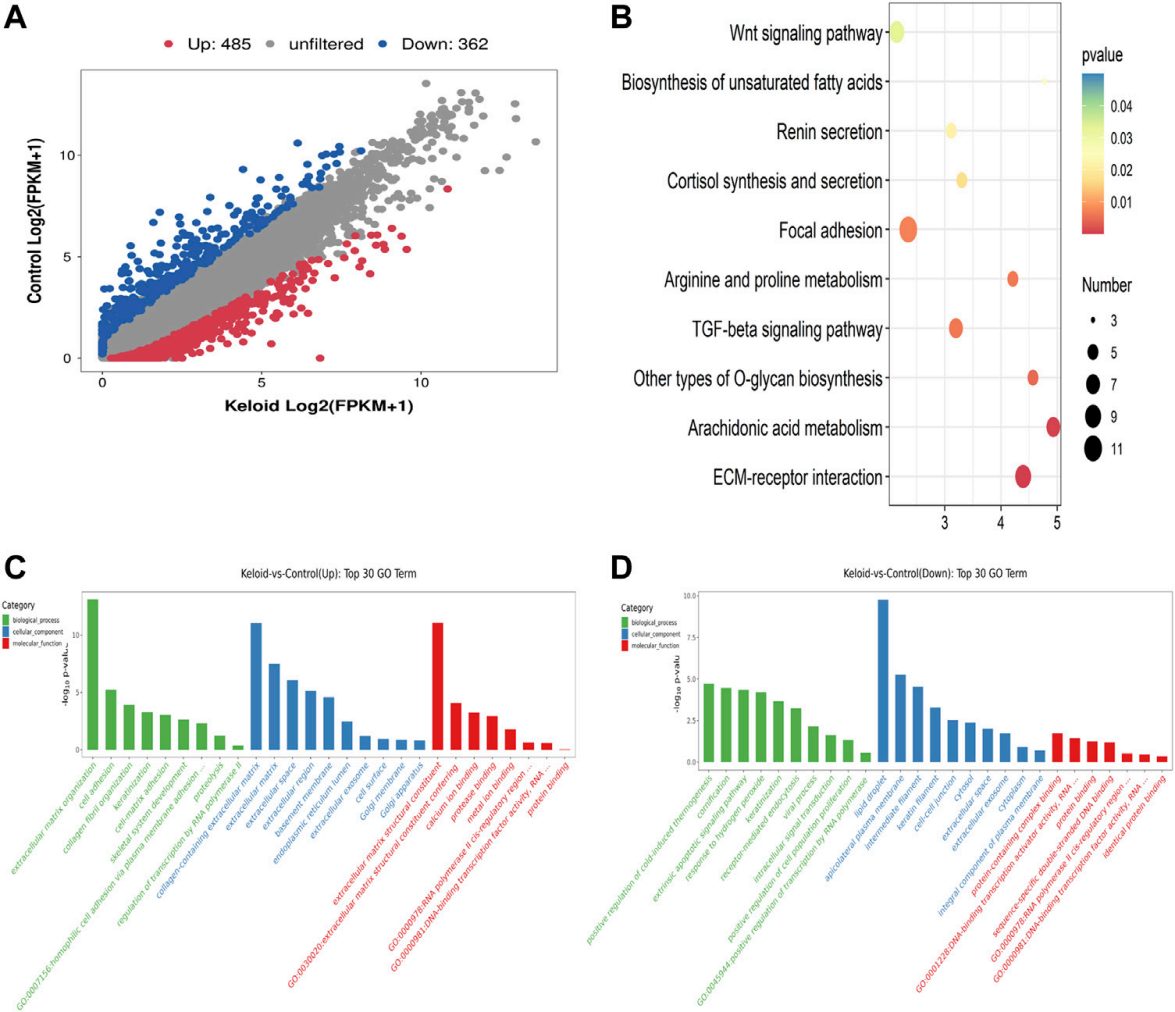
Differentially expressed genes (DEGs), KEGG, and GO items in keloid. **(A)** Scatter plot of the RNA-seq data between two groups. **(B)** Top ten pathways of the increased mRNA in the keloid samples compared with those in NS samples. **(C)** Overview of the distributions of the upregulated genes in various GO categories including biological process (BP), cellular component (CC), and molecular function (MF). **(D)** Overview of the distributions of the downregulated genes in BP, CC, and MF.

The m^6^A peaks were **highly** enriched within 3′-UTR close to the coding sequence (CDS) region in the mRNA (Figure 2A). Visualization of the distribution of the transcriptome-wide m^6^A peaks across the 24 chromosomes was performed on the normal skin and keloid tissue. The results showed that the overlapping peaks between the two groups and the unique peaks of keloid were distributed on each chromosome, and the distribution patterns coincided with the gene content density (Figure 2B). Most of the hypomethylated peaks were enriched in chromosomes 1 (572 peaks), 17 (431 peaks), and 19 (510 peaks). Intriguingly, most of the hypermethylated peaks were also enriched in chromosomes 1 (107 peaks), 17 (79 peaks), and 19 (75 peaks) (Figure 2B). In addition, the hypomethylated peaks with the largest widths were distributed on chromosomes 1, 11, and 19, while hypermethylated peaks with the largest widths were distributed on chromosomes 6, 12, and 10 (Figure 2B). Figure 2C shows the top three high-conservative sequence motifs in the two groups. The identified m^6^A peaks in the keloid group were most abundant in consensus sequences of RRACH (*p*-value: 1.5E-019) (R represents A or G, and H represents A, T, or C) (Figure 2C), which proves that typical sequence motifs are related to m^6^A (Zhao et al., 2017). Kyoto Encyclopedia of Genes and Genomes pathway analysis was also conducted for m^6^A-modified genes in the keloid group and the NS group. The genes with m^6^A-methylated modification in keloid were mainly associated with the Wnt signaling pathway, TNF signaling pathway, and TGF-beta signaling pathway, which were related to the biological process of proliferation, differentiation, and migration (Figure 2D). Our result is consistent with the previous studies, which reported that the Wnt signaling pathway was activated in keloid in comparison to the normal skin (Chua et al., 2011; Lee et al., 2015). Moreover, the top 10 pathways between keloid and normal skin were almost overlapped, while the signaling pathways of Wnt, Notch, NF-kappa B, and Hippo were specifically clustered in the keloid m^6^A-modified gene pool. Since the candidate gene numbers in the NF-kappa B and Hippo pathways were less than 100 (Supplementary Table S1) and the Wnt signaling pathway was also enriched in the top 10 upregulated KEGG pathways of the DEGs between keloid and normal skin (Figure 3B), it indicated that the upregulated Wnt pathway may be induced by high m^6^A modification.

### RNA-sequencing analysis of the keloid group and the NS group

Compared with the NS group, 847 genes were significantly changed in the keloid group, of which 485 genes were upregulated and 362 genes were downregulated (fold change >2, *p* < 0.05) (Figure 3A). We further conducted GO and KEGG enrichment analyses for these differentially expressed genes (DEGs). The top ten pathways of the increased mRNA modification in the keloid samples compared with NS samples are listed in Figure 3B. KEGG analysis identified a bunch of significantly enriched pathways related to proliferation and migration, such as the Wnt signaling pathway and TGF-beta signaling pathway (Figure 3B). The upregulated Wnt signaling pathway was consistent with the hyper-m^6^A-modified genes signaling pathway (Figure 2D). Figures 3C,D provide an overview of the distributions of the differentially expressed genes in various GO categories including biological process (BP), cellular component (CC), and molecular function (MF). Abnormally upregulated genes were obviously associated with the extracellular matrix organization and collagen fibril organization (ontology: biological process), collagen-containing extracellular matrix (ontology: cellular component), and extracellular matrix structural constituent (ontology: molecular function) (Figure 3C). Abnormally downregulated genes were significantly enriched in the detoxification of copper ions and cellular response to copper ions (ontology: BP), extracellular space (ontology: CC), and oxygen binding and carrier activity (ontology: MF).

### The combined analysis of MeRIP-seq and RNA-seq with the verification of the Wnt/*β*-catenin signaling pathway

**FIGURE 4:**
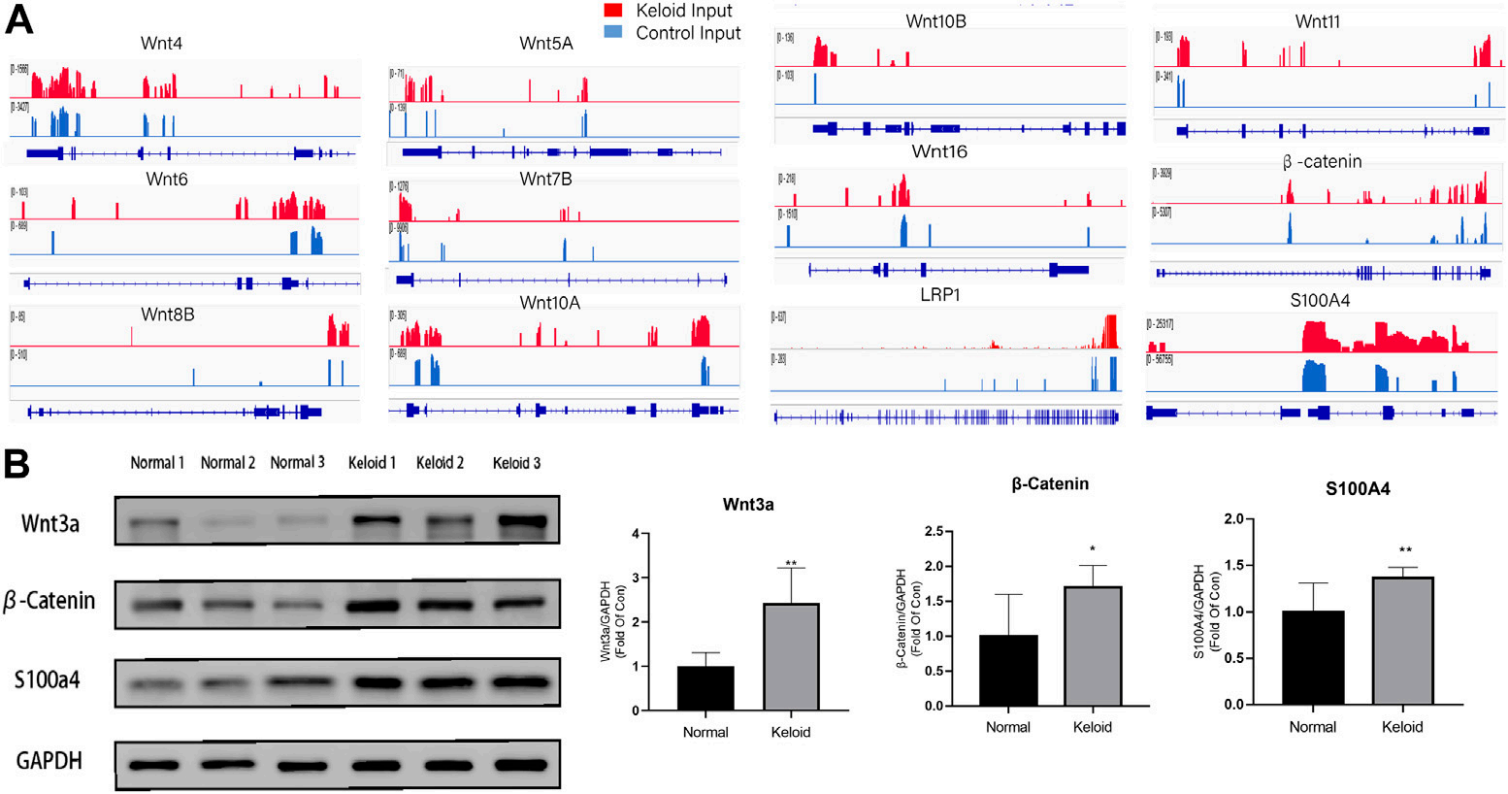
Difference between the m^6^A modification and the activation of the Wnt/*β*-catenin pathway between the two groups. **(A)** m^6^A modification of LRP1, Wnt isoforms, and *β*-Catenin in keloid compared with that in NS in the combined analysis of MeRIP-seq and RNA-seq. **(B)** Protein expression of the Wnt/*β*-catenin/S100A4 proteins in keloid compared with that in NS. *p < 0.05, **p < 0.01.

As the Wnt/*β*-catenin pathway is closely related to the migration and proliferation of fibroblast and tumor cells, we furthermore investigated the pathway with the combined analysis of MeRIP-seq and RNA-seq. The mRNAs of LRP1, isoforms of Wnt, and *β*-catenin in the Wnt signaling pathway were visualized using the IGV tracks (Figure 4A). The distributions of the peak regions in these mRNAs were displayed to be largely located in the 3′-UTR (Figure 4A). Moreover, these genes have more m^6^A distribution in the keloid than those in normal skin tissue. Three samples were randomly selected from 14 samples in two groups to determine the activation of Wnt/*β*-catenin in the keloid group. Since S100A4 is both the target gene of the Wnt/*β*-catenin signaling pathway and the biomarker of fibroblast, we verified the protein of Wnt, *β*-catenin, and S100A4 using Western blotting. The results showed that the expression of Wnt3a, *β*-catenin, and S100A4 proteins in keloid was significantly higher than that in normal skin (Figure 4B, *p* < 0.05). Such a result was consistent with our presumption that the hyper-m^6^A-modified highly expressed Wnt/*β*-catenin/S100A4 pathway played a role in the pathogenesis of keloid.

### The hyper-m^6^A-conditioned fibroblasts contribute to the formation of keloid

**FIGURE 5:**
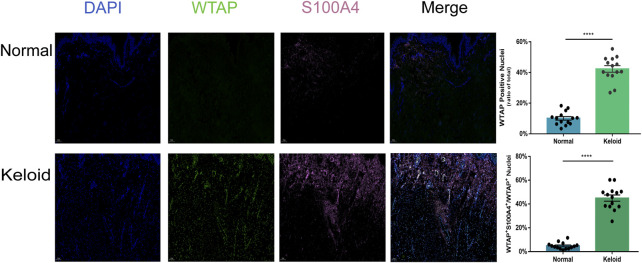
Positive effects of hypermethylation on the expression of fibroblasts in keloid. Three-color immunofluorescence confocal images were obtained for WTAP (green), S100A4 (pink), and nuclei (blue) in normal skin and keloid. The images were merged at last. The two bars show that the proportion of the cells with WTAP staining and the cells with both positive staining of WTAP and S100A4 in keloid were significantly higher than those in normal skin. ****p < 0.0001.

As the activated fibroblast promotes the pathological change of keloid by overproducing the collagen subcutaneously, we furthermore investigated the epitranscriptomic modification of fibroblast in the keloid tissue. There was a higher fluorescence intensity of S100A4 in the keloid than in the normal skin (Figure 5), which meant accumulated fibroblast contributes to the pathogenesis of keloid (Manetti et al., 2017). By overlapping WTAP and S100A4 staining, we found that almost all S100A4 positively stained fibroblasts had WTAP-stained nuclei (Figure 5). Since WTAP interacts with METTL3 and METTL14 and is required for their localization into nuclear speckles enriched with pre-mRNA processing factors and for the catalytic activity of m^6^A methyltransferase *in vivo* (Ping et al., 2014), it presented the fact that the WTAP^+^ fibroblasts regulates the pre-mRNA processing and promotes the m^6^A methyltransferase activity during the formation of keloid. The two bars of Figure 5 showed that the ratio of WTAP positively stained nuclei in keloid was significantly higher than that in normal skin, and the ratio of the cells with both positive staining of WTAP and S100A4 in keloid was significantly higher than that in normal skin (Figure 5, ****p < 0.0001). The results showed that the dermal fibroblasts under a hyper-m^6^A condition contribute to the pathogenesis of keloid.

## Discussion

Keloid is one of the benign fibro-proliferative tumors with excessive fibrosis deposition (Wang et al., 2020). It is prone to recurrence with the current treatments on the keloid, such as operational excision and intralesional injection with drugs (Gold et al., 2014). More and more evidence showed that keloid is different from hypertrophic scar and normal scar since it has a different clinical feather (Burd and Huang, 2005) and relative progressive mechanisms (Limandjaja et al., 2020). While hypertrophic scars grow within the borders of the original wound and eventually grow smaller, keloids grow beyond the original wound borders and do not grow smaller on their own, thus making it seriously affecting the personal appearance or even causing dysfunction if they formed in the joints. Accumulative studies reported that the formation of the keloid was closely associated with the CD34^−^/*α*-SMA+/p16 + phenotype along with strong immunoreactivity for p16 (Limandjaja et al., 2020), abnormal signaling regulation pathways in the fibroblast such as noncoding RNA (Gao et al., 2022; Kuai and Jian, 2022; Zhu et al., 2022) or exosome secretion (Shen et al., 2022). Moreover, studies found that keloids could be divided into high-risk and low-risk groups with differences in immunity, m6A methylation, and pyroptosis (Xie et al., 2022). However, few studies focused on the epitranscriptomic feathers of the keloid. Since the epitranscriptomic modification affects most phenotypes of diseases, it is significant to have a comprehensive understanding of the m^6^A-modifying characteristics of keloid. As one of the most common epitranscriptomic modifications in mRNA, N^6^-methyladenosine (m^6^A) occurs in roughly 25% of transcripts at the genome-wide level (Lan et al., 2019). Such modification can alter the fate of RNA by changing mRNA stability, splicing, transport, localization, and translation (Wang et al., 2014), which affects various biological processes including differentiation, self-renewal, and proliferation (Deng et al., 2018). In the epitranscriptomic study of hypertrophic scar, Liu et al. (2021) showed that the fibrosis-related genes had hyper-m^6^A modification levels in the scar tissue. However, it remains unclear that which type of cell under the hyper-m^6^A condition promotes the formation of the scar-, and the protein level of the associated pathway between the scar tissue and normal skin tissue (Liu et al., 2021). As for the keloid, the relationship between m^6^A modification and keloid has not been investigated yet. The present study first presented the epitranscriptomic profile of the keloid and verified the underlying mechanism with experimental tests, which determines that the hyper-m^6^A-modified fibroblasts contribute to the pathogenesis of keloid.

In the study, the proteins levels of METTL3 and WTAP in the keloid were significantly higher than those in the NS, and there is no difference in METTL14, FTO, and ALKBH5 (the demethylase) between the keloid and normal skin. Therefore, the keloid was under the hyper-m^6^A-methylated condition compared with the normal skin (Figure 1A). The MeRIP-seq furthermore showed that there are 21,020 m^6^A unique peaks and 6,573 unique m^6^A genes in the keloid. In the keloid tissue, we also found that the unique peaks of keloid were distributed on each chromosome, and both the hypomethylated peaks and hypermethylated peaks were enriched in the same chromosomes (1, 17, and 19). In addition, we performed the KEGG pathway analysis to investigate the potential function of m^6^A-modified transcripts in each group. The top one related pathway in keloid was the Wnt signaling pathway, which plays a role in the cell–cell communication system that is important for stem cell renewal, proliferation, and differentiation (Steinhart and Angers, 2018). Moreover, the Wnt signaling pathway was also ranked as the first of the elevated pathways transcriptionally in keloid than in NS tissues. The consistent change suggests that the hyper-m^6^A-modified Wnt signaling pathway promotes its higher transcriptional level in the keloid than in the normal tissue, thus making it possible for the subsequent activation of the Wnt signaling pathway with increasing the proteins levels of Wnt3a, *β*-catenin, and S100A4 in the keloid. Moreover, the immunofluorescence staining result showed that S100A4 and WTAP were overlapped in the same cells in the keloid. Such a phenomenon presented the fact that the accumulated fibroblasts were under a hyper-m^6^A condition in the keloid. Moreover, it seems that the Wnt/*β*-catenin signaling pathway was mainly activated in the fibroblasts since S100A4 is also the target gene of the signaling pathway.

In the present study, the sample size is limited, and the normal skin samples should be selected adjacent to the keloid. Follow-up studies require a larger sample size and the corresponding normal skin samples. In addition, functional experiments are needed to confirm the influence of m^6^A RNA modifications upon gene expression in keloid. To further determine the regulatory effect of m^6^A RNA modification on the pathogenesis of keloid, *in vivo* and *in vitro* tests with the knocking-out or overexpressing METTL3 and WTAP fibroblasts should also be carried out.

## Conclusion

In conclusion, we first revealed the global m^6^A modification pattern in the keloid samples and in the normal skin samples. From all aforementioned data, we draw a conclusion that the fibroblasts were under a cellular hyper-m^6^A-methylated condition, and the hyper-m^6^A-modified highly expressed Wnt/*β*-catenin/S100A4 pathway in the dermal fibroblasts might promote the pathogenesis of keloid.

## Data Availability

The original contributions presented in the study are publicly available. This data can be found here: Gene Expression Omnibus GSE202851 at: https://www.ncbi.nlm.nih.gov/geo/query/acc.cgi?acc=GSE202851; Gene Expression Omnibus GSE202855 at: https://www.ncbi.nlm.nih.gov/geo/query/acc.cgi?acc=GSE202855; Gene Expression Omnibus GSE202861 at: https://www.ncbi.nlm.nih.gov/geo/query/acc.cgi?acc=GSE202861.
