# Corrigendum: The m^6^A-methylated mRNA pattern and the activation of the Wnt signaling pathway under the hyper-m^6^A-modifying condition in the keloid

**DOI:** 10.3389/fcell.2023.1144733

**Published:** 2023-01-31

**Authors:** Can-Xiang Lin, Zhi-Jing Chen, Qi-Lin Peng, Ke-Rong Xiang, Du-Qing Xiao, Ruo-Xi Chen, Taixing Cui, Yue-Sheng Huang, Hong-Wei Liu

**Affiliations:** ^1^ Department of Plastic Surgery of the First Affiliated Hospital of Jinan University, Institute of New Technology of Plastic Surgery of Jinan University, Key Laboratory of Regenerative Medicine of Ministry of Education, Guangzhou, China; ^2^ The Research Center of Medicine, Sun Yat-Sen Memorial Hospital, Sun Yat-Sen University, Guangzhou, China; ^3^ Department of Thoracic surgery, The First Affiliated Hospital of Jinan University, Guangzhou, China; ^4^ Department of Cell Biology and Anatomy, University of South Carolina School of Medicine, Columbia, SC, United States; ^5^ Department of Wound Repair, Institute of Wound Repair and Regeneration Medicine, Southern University of Science and Technology Hospital, Southern University of Science and Technology School of Medicine, Shenzhen, China

**Keywords:** Keloid, m^6^A modification, fibroblasts, the Wnt signaling pathway, RNA sequencing

In the published article, there was an error in affiliation 1. Instead of “Key Laboratory of Regenerative Medicine, Ministry of Education, Department of Plastic Surgery, The First Affiliated Hospital of Jinan University, Guangzhou, China”, it should be “Department of Plastic Surgery of the First Affiliated Hospital of Jinan University, Institute of New Technology of Plastic Surgery of Jinan University, Key Laboratory of Regenerative Medicine of Ministry of Education, Guangzhou, China.”

The authors apologize for this error and state that this does not change the scientific conclusions of the article in any way. The original article has been updated.

