# Supplementary material for: The m6A-methylated mRNA pattern and the activation of the Wnt signaling pathway under the hyper-m6A-modifying condition in the keloid
Source: Front Cell Dev Biol. 2022 Oct 3;10:947337. doi: 10.3389/fcell.2022.947337 (PMC9574062; doi:10.3389/fcell.2022.947337)
Supplement: Supplementary file 1 [file DataSheet1.docx]

Supplementary Table 1. Top 10 KEGG Pathway of the m6A-modified Genes in Each Group


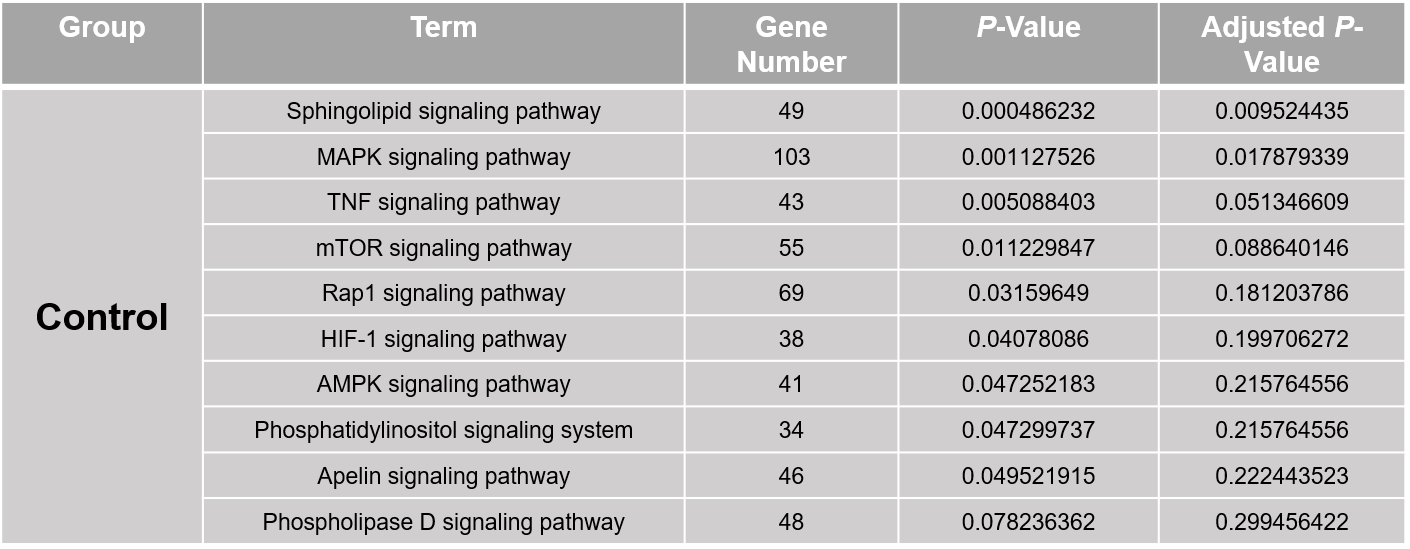


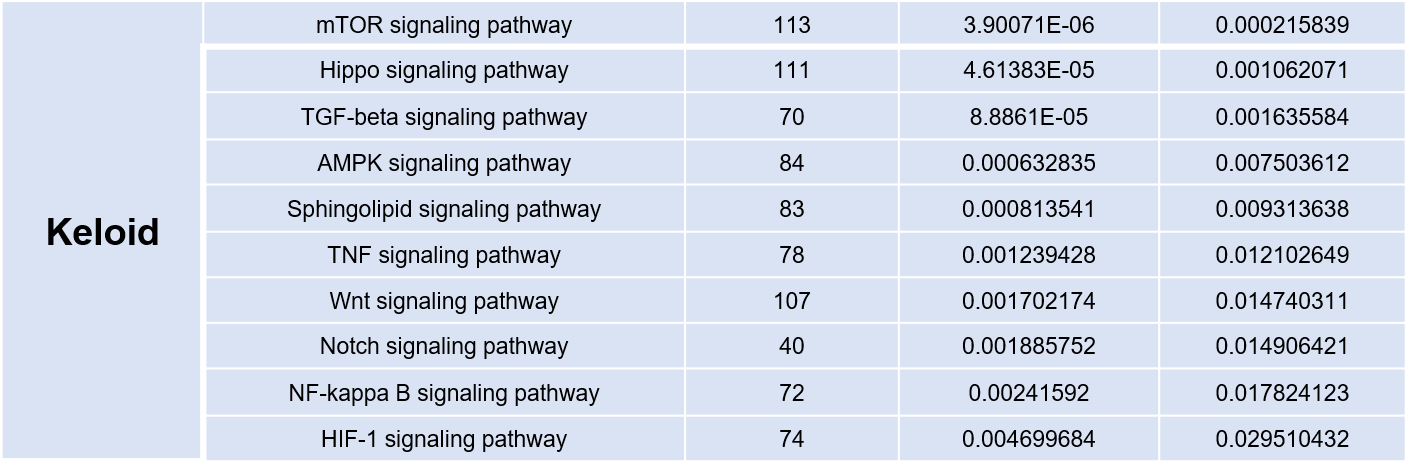


Supplementary Table 2. Top 10 Up-Regulated KEGG Pathway Enriched in the Normal Skin

| **Term** | **Gene Number** | ***P*-Value** | **Adjusted *P*-Value** |
| --- | --- | --- | --- |
| Wnt signaling pathway | 8 | 0.032981306 | 0.408968197 |
| Biosynthesis of unsaturated fatty acids | 3 | 0.024067855 | 0.352432372 |
| Renin secretion | 5 | 0.021888238 | 0.352432372 |
| Cortisol synthesis and secretion | 5 | 0.017318572 | 0.321829325 |
| Focal adhesion | 11 | 0.00714567 | 0.196902893 |
| Arginine and proline metabolism | 5 | 0.00638522 | 0.196902893 |
| TGF-beta signaling pathway | 7 | 0.006146686 | 0.196902893 |
| Other types of O-glycan biosynthesis | 5 | 0.00449234 | 0.18568339 |
| Arachidonic acid metabolism | 7 | 0.000497602 | 0.041135136 |
| ECM-receptor interaction | 9 | 0.000193009 | 0.023933154 |
